# Acute abrin poisoning treated with continuous renal replacement therapy and hemoperfusion successfully: A case report

**DOI:** 10.1097/MD.0000000000007423

**Published:** 2017-07-07

**Authors:** Jiliang Huang, Wenbin Zhang, Xin Li, Shufen Feng, Gang Ye, Hongcheng Wei, Xiaobing Gong

**Affiliations:** a Department of Gastroenterology; b Department of Gastrointestinal Surgery, The First Affiliated Hospital of Jinan University, Guangzhou, Guangdong, China.

**Keywords:** abrin, *Abrus precatorius*, continuous renal replacement therapy, hemoperfusion, poisoning

## Abstract

**Rationale:**

Abrin is a highly toxic protein obtained from the seeds of *Abrus precatorius*, but poisoning due to ingestion of *A precatorius* is extremely rare in China.

**Patient concerns:**

A 16-year-old girl, perfectly healthy before, was admitted to the department of gastroenterology owing to intentional ingestion of 10 crushed *A precatorius* seeds, with multiple episodes of somnolent and anxious mental status, vomiting, abdominal pain, diarrhea, hematochezia, and hematuria.

**Diagnosis:**

Acute abrin poisoning.

**Interventions:**

We immediately took effective measures including gastric lavage, purgation, gastric acid suppression by proton pump inhibitor (PPI), liver protection, hemostasis, blood volume and electrolytes resuscitation, continuous renal replacement therapy (CRRT), and hemoperfusion (HP).

**Outcomes:**

Her unwell mental status was improved to the point at which she became conscious and relaxed. The symptoms of vomiting, abdominal pain, diarrhea, hematochezia, and hematuria disappeared gradually. The girl eventually made an excellent recovery with no complications at her 3-month follow-up.

**Lessons:**

The combination of CRRT and HP is an efficient measure in the treatment of abrin poisoning for which there is no specific antidote. This is the first reported case of an abrin poisoning patient successfully treated by CRRT plus HP. Our experience will be useful to other physicians in managing patients of acute abrin poisoning in the future.

## 1 Introduction

**Figure 1:**
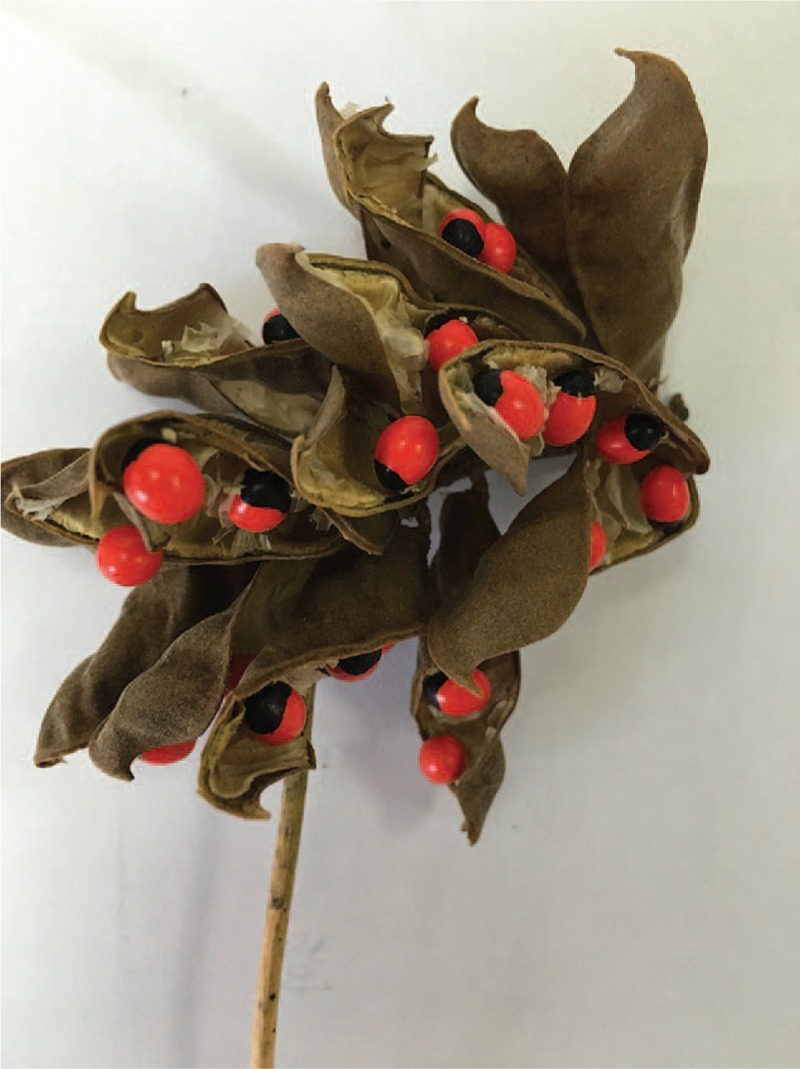
The seeds of *A precatorius* are shiny and red, with a black circular tip.

*Abrus precatorius* is one of the deadliest poisons in the world, known commonly as rosary peas, jequirity bean, and crab's eye (Fig. 1). Abrin is a highly toxic protein obtained from the seeds of *A precatorius*, with an estimated human fatal dose of 0.1 to 1 μg/kg, and has caused death after ingestion of 1 to 2 crushed seeds.^[1]^ Acute hemorrhagic gastroenteritis with erosions is characteristic symptom of abrin poisoning, presenting as vomiting and watery diarrhea coming first, later bloody diarrhea and black stools.^[2]^ Poisoning due to ingestion of *A precatorius* is extremely rare in China. Here, we report a case of abrin poisoning in a 16-year-old girl who was treated successfully with continuous renal replacement therapy (CRRT) and hemoperfusion (HP).

## 2 Case report

A 16-year-old girl (perfectly healthy before, 40 kg body weight) was admitted to the department of gastroenterology owing to intentional ingestion of 10 crushed seeds of *A precatorius* (approximate weight 5 g) at 5 p.m. on April 12, 2016. Following ingestion of the seeds, the girl developed multiple symptoms of nausea and vomiting. Two hours after ingestion, she emerged abdominal pain (persistent pain, obviously in upper and middle abdomen) and diarrhea (watery diarrhea, approximately 2 times/h). After admission, the girl started defecating liquid black stools which turned into bloody later; her urine presented in red color and urine output decreased (less than 30 mL/h).

On physical examination, she looked unwell, with a temperature of 37.0°C, heart rate of 112 beats/min, respiratory rate of 30 breaths/min, blood pressure of 85/48 mm Hg, and oxygen saturation of 90%. The girl had a somnolent and anxious mental status. Her whole abdomen had tenderness, especially in the upper and middle abdomen, but without abdominal muscle tension or rebound tenderness. The bowel sound was active (about 10 times/min).

Laboratory tests showed white blood cell 14.97 × 10^9^ cells/L, neutrophil 13.60 × 10^9^ cells/L, hemoglobin 129 g/L, creatine kinase (CK) 429 U/L, and glucose 6.84 mmol/L. The urine routine test revealed: leukocyte 51 LEU/μL, erythrocyte 90 ERY/μL, and glucose 2.1 mmol/L. Stool routine test indicated: leukocyte 2 WBC/HP, erythrocyte 26 RBC/HP, and fecal occult blood test (+). Other tests, including liver and renal function, coagulation function, serum electrolytes, and creatine kinase MB isoenzyme (CK-MB), were normal. According to the girl's symptoms and these results, we confirmed she was suffering from *A precatorius* poisoning.

**Figure 2:**
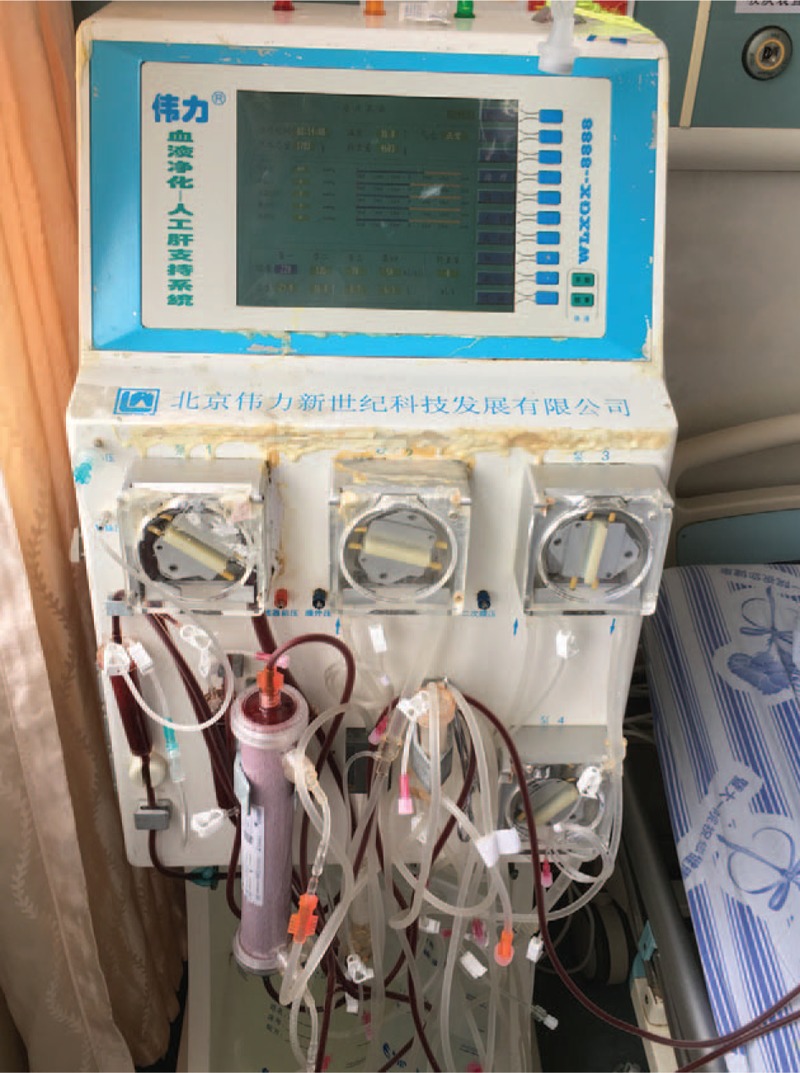
A nonbiological artificial liver (WLXGX-8888 Weili Artificial Liver Support System; Beijing Weili New Century Technology Development Co. Ltd, Beijing, China).

We immediately performed cross matching and placed an intravenous catheter. In addition, we closely monitored vital signs and treated the patient with gastric lavage, purgation, gastric acid suppression by PPI, liver protection, blood volume and electrolytes resuscitation, and hemostasis. Meanwhile, the patient underwent CRRT in combination with HP through an emergency indwelling dual-lumen catheter placed in the right subclavian vein, using a nonbiological artificial liver (WLXGX-8888 Weili Artificial Liver Support System; Beijing Weili New Century Technology Development Co. Ltd, Beijing, China) (Fig. 2). CRRT was administered for 72 hours and was performed continuous venovenous hemofiltration (CVVH) with a Polyflux 140H capillary dialyzer (Gambro Dialysatoren GmbH, Germany; synthetic high flux membrane, with a membrane area of 1.4 m^2^). The prescription of replacement fluid (140 mmol/L Na^+^, 105 mmol/L Cl^−^, 4.2 mmol/L K^+^, 2.4 mmol/L Ca^2+^, 0.8 mmol/L Mg^2+^, 39 mmol/L HCO3^−^ and 7.3 mmol/L glucose) was transfused using a predilution method. The CVVH used a blood flow of 180 mL/min, a replacement fluid rate of 3 L/h and an ultrafiltration rate of 50 mL/h. Hemoperfusion was carried out for 2 hours from 13:35 to 15:35 on April 13 using a disposable HA330-II resin hemoperfusion cartridge (Zhuhai Jafron Biomedical Co., Ltd., Guangdong, China), with a blood flow of 180 mL/min. We closely kept monitoring related parameters until the patient was fully recovered, and basic laboratory tests according to time elapsed after admission are shown in Table 1.

**Table 1:**
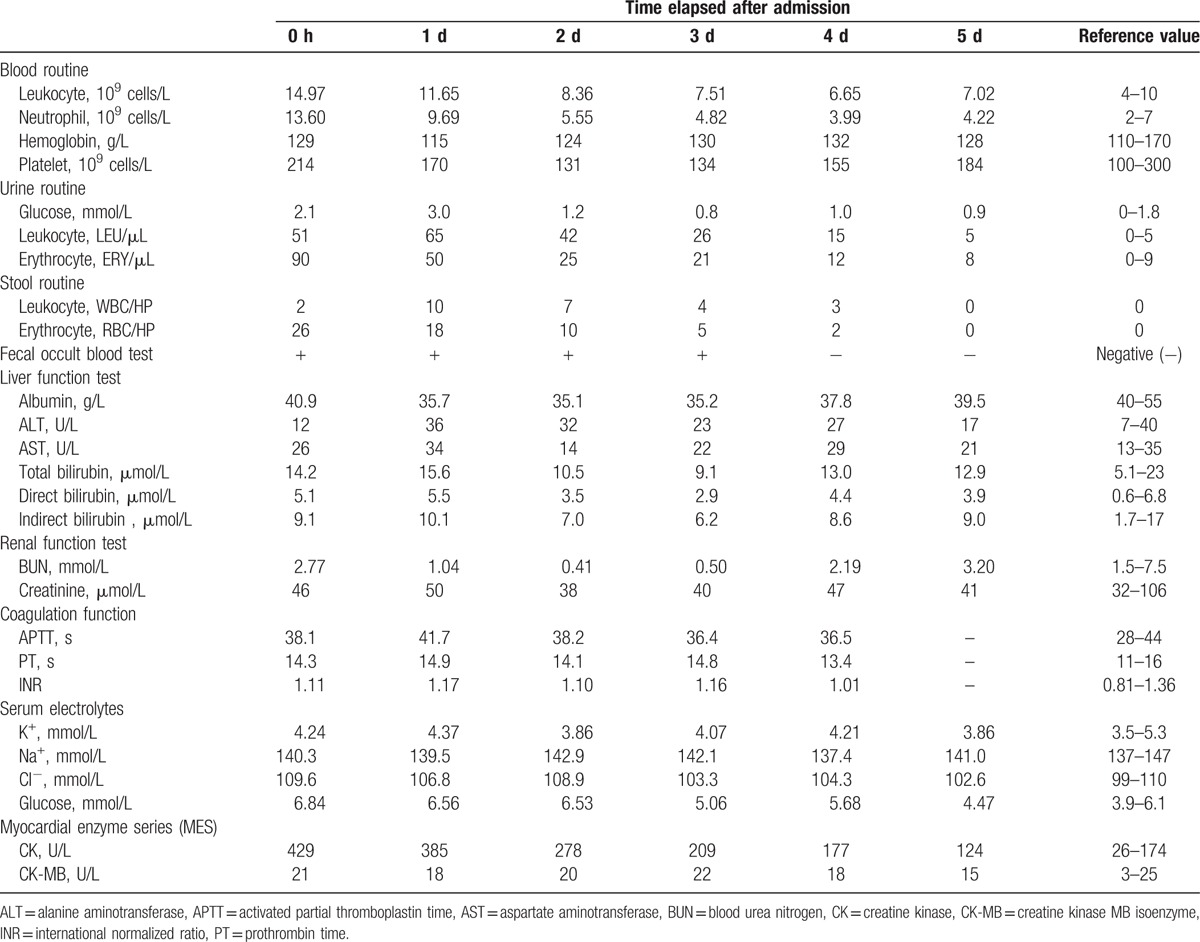
Laboratory test.

The patient's condition started improving after the combined therapy of CRRT and HP, including that heart rate fluctuated at 70 to 90 beats/min, blood pressure was stable and in the range 100–120/55–75 mm Hg, oxygen saturation maintained over 98%. Her unwell mental status was improved to the point at which she became conscious and relaxed. The symptoms of vomiting, abdominal pain, bloody stools, gross hematuria, dizziness, and fatigue were ameliorated gradually. Urine output increased to 60 mL/h after 3 days. At the 5th day after admission, we checked the blood routine, urine routine, stool routine and occult blood test, liver and renal function, coagulation function, serum electrolytes, and myocardial enzyme series for the patient, and all parameters returned normal. The girl eventually made an excellent recovery with no sequelae at her 3-month follow-up.

## 3 Discussion

Abrin is a toxalbumin with a molecular weight of 65 kDa, which accounts for approximately 0.075% by weight of the *A precatorius*.^[1,3]^ Abrin has 2 subunits, an A and B chain covalently linked through a disulfide bridge. The abrin A chain with a molecular weight of approximately 30 kDa acts on the 60 s ribosomal subunit to inhibit elongation factor EF-1 and EF-2, so that preventing protein synthesis and causing cell death.^[4,5]^ The abrin B chain (molecular weight 35 kDa) promotes binding to cell-surface receptors and permits the penetration of the A chain into cells.^[4,6]^

Intact *A precatorius* often pass through gastrointestinal (GI) tract without harm, whereas ingestion of crushed seeds will be of great toxicity to human body.^[2]^ Even if abrin is significantly digested and poorly absorbed, the undigested abrin is still sufficient to result in severe complications.^[1]^ Most reported cases of abrin poisoning predominantly cause gastrointestinal (GI) toxicity, such as vomiting, abdominal pain, diarrhea, hematochezia, and so on.^[7–9]^ After ingestion of rosary peas, the initial symptoms may occur in 6 hours, but sometimes it will be delayed for 1 to 3 days.^[2,7]^ The severe vomiting and diarrhea will cause water-electrolyte metabolism disorder and acid–base balance disorder, or even lead to more severe sequelae such as hypovolemic shock, liver and kidney failure, and coagulation disorder.

CRRT refers to a treatment which applied 24-hour continuous blood purification and is usually used to treat the acute renal failure, certain drugs, or toxicants poisoning. It has several advantages over intermittent dialysis. The membranes used in CRRT are ordinarily more permeable than intermittent hemodialysis membranes. Most hemodialysis membranes allow for the clearance of molecules up to 1 kDa, but CRRT membranes allow for the clearance of molecules as large as 20 kDa.^[10]^ Besides, CRRT will not significantly influence hemodynamics and can be used for hemodynamically unstable patients to maintain blood pressure.^[11,12]^ However, the distinct drawback of CRRT is the relatively slower clearance rates for toxicants poisoning compared with standard intermittent hemodialysis. Moreover, CRRT usually need the application of heparin, which can increase the risk of bleeding and electrolyte disturbances.^[11]^

Hemoperfusion is an extracorporeal treatment of filtering the blood to remove toxic metabolites or exogenous toxin based on adsorption, and it is usually used for the treatment of acute poisonings.^[13]^ The toxins removal rate of hemoperfusion is better than that of conventional hemodialysis. HP could effectively absorb protein- and lipid-bound toxins and reduce the concentration of abrin in the blood, thereby decreasing the severity of toxicity and duration of poisoning.^[13]^

In this patient, the girl (40 kg body weight) intentionally ingested 10 crushed *A precatorius* seeds (approximate weight 5 g). Therefore, her estimated dose of ingested abrin was 93.75 μg/kg and it obviously exceeded the human fatal dose. The conventional treatments including gastric lavage, purgation, gastric acid suppression by PPI, liver protection, blood volume and electrolytes resuscitation, and hemostasis were performed immediately after admission. Considering the severity of abrin poisoning and hemodynamic instability, traditional hemodialysis was not initially considered. We simultaneously used CRRT plus HP to eliminate the toxins and their toxic products. Abrin has a high molecular weight (approximately 65 kDa) and it is not therefore easily cleared by CRRT. But CRRT can be run 24 hours a day, which could slowly and continuously remove low and middle molecule solute including creatinine, low molecular weight degradation products of abrin, and inflammatory mediators over a long period, and it will not significantly influence hemodynamics. Hemoperfusion may play a major role in the abrin removal and pave the way for the following CRRT and compensate for the drawback of CRRT; however, it cannot be ignored that CRRT removed low molecular weight degradation products of abrin, creatinine, and inflammatory mediators, maintained hemodynamic stability and improved the internal environment. Before acute liver and kidney injury appear, the combined therapy of CRRT and HP could facilitate toxins clearance and reduce toxins concentration in the body, which will finally prevent exacerbation and reduce mortality.

In this case, we have learned the following experience: (1) gastric lavage and purgation could remove toxin remnants from digestive track, thus further absorption will be reduced. (2) Establish a venous channel in time and keep monitoring vital signs closely. This is the key to our successful rescue. (3) Maintain effective circulating blood volume and ensure blood perfusion of vital organs (heart, brain, liver, kidney, etc.). (4) Adopt PPI in the early to handle gastrointestinal tract hemorrhage. PPI can increase the gastric pH value, and thus promote platelet aggregation, facilitate fibrin clot formation, and avoid lysis of blood clot. (5) Facilitate toxins decontamination from blood thoroughly. The combination of CRRT and hemoperfusion may exert a beneficial effect and realize effective and continuous toxicants clearance; thus, toxicants rebound could be suppressed and patient's condition could be promoted.

In conclusion, acute abrin poisoning can be treated with CRRT and hemoperfusion successfully. Blood purification is an effective measure to eliminate toxicants from the body in acute abrin poisoning. After reviewing the related literatures, we find that the combined application of CRRT and hemoperfusion in the treatment of abrin poisoning have not been reported within China and abroad. Our experience will be useful to other physicians in managing patients of acute abrin poisoning in the future.

## Acknowledgments

The authors thank Dongli Wang and Kevin Chao from the Department of Public Health in California for polishing the language of this manuscript.
